# Targeting RPRD1B overcomes chemoresistance in gastric cancer by suppressing the TOPBP1-mediated DNA damage repair pathway

**DOI:** 10.1007/s13402-026-01227-0

**Published:** 2026-08-02

**Authors:** Jian Sheng, Jianghua Li, Chenxi Cao, Xiaorong Liu, Chunhua He, Mingjian Fei, Bin Wu, Xiangli Li, Chundong Hu, Shumin Liu, Yahui Lv

**Affiliations:** 1 School of Basic Medical Sciences, Tsinghua University, Beijing, China; 2 Department of Research and Teaching, The Second Affiliated Hospital of Jiaxing University, Jiaxing, China; 3 Department of Surgical Oncology, The Second Affiliated Hospital of Jiaxing University, Jiaxing, China; 4 Department of Hepatobiliary Surgery, The Second Affiliated Hospital of Jiaxing University, Jiaxing, China; 5 Department of Pathology, The Second Affiliated Hospital of Jiaxing University, Jiaxing, China; 6 Department of Pathology, First Hospital of Tsinghua University, Jiaxing, China; 7 Department of Respiratory Medicine, Tangdu Hospital, Fourth Military Medical University, Xian, China; 8 Department of Oncology, The Second Affiliated Hospital of Jiaxing University, Jiaxing, China

**Keywords:** 5-FU, RPRD1B, DNA damage repair, AAV

## Abstract

**Background:**

Despite the widespread adoption of 5-fluorouracil (5-FU)-based regimens as first-line therapy for gastric cancer, a substantial number of patients develop innate or acquired resistance, highlighting the critical need to identify its underlying molecular drivers. RPRD1B (CREPT), a gene frequently overexpressed in GC, has been clinically associated with advanced tumor stage and poor prognosis. Functionally, RPRD1B promotes aggressive tumorigenic phenotypes by accelerating cell-cycle progression, potentiating proliferative signaling, and enhancing the migratory and invasive capacities of cancer cells. However, its functional role in mediating chemotherapy resistance has not been elucidated.

**Methods:**

We established 5-FU-resistant gastric cancer cell lines (from AGS/MGC803 parents via stepwise drug exposure) to study RPRD1B’s mechanism, and developed an AAV-based system to therapeutically target RPRD1B to overcome 5-FU resistance.

**Results:**

In this study, we demonstrated that GC patients with high tumor expression of RPRD1B (CREPT) exhibited a poor response to 5-fluorouracil (5-FU)-based chemotherapy. Furthermore, RPRD1B expression was positively correlated with the expression of TOPBP1, a critical DNA damage response and repair effector. Mechanistically, RPRD1B transcriptionally upregulates TOPBP1 by recruiting RNA polymerase II to its promoter, thereby enhancing DNA damage repair. Using an AAV-delivered shRNA to knockdown RPRD1B in a nude mouse xenograft model, we effectively overcame 5-FU resistance in gastric tumors in vivo.

**Conclusions:**

Our findings identify RPRD1B as a promising therapeutic target for reversing chemoresistance in gastric cancer.

**Supplementary Information:**

The online version contains supplementary material available at 10.1007/s13402-026-01227-0.

## Introduction

Gastric cancer is a common malignant tumor remains a significant global health burden due to its high incidence and mortality rates [1]. Gastric cancer is a leading cause of cancer-related deaths worldwide. Chemotherapy regimens based on 5-fluorouracil (5-FU) form a cornerstone of treatment for the advanced stages of the disease [2–4]. However, the clinical use of this drug is largely limited by the emergence of resistance, which may be attributed to a range of mechanisms, such as impaired drug accumulation due to altered transport, accelerated drug metabolism, or target site mutations [5]. Currently, oxaliplatin and irinotecan are used to overcome 5-FU resistance. Oxaliplatin, a platinum-based DNA-damaging agent, demonstrates enhanced efficacy when combined with 5-FU/LV, significantly improving response and survival. Irinotecan, a topoisomerase I inhibitor, is effective in first- and second-line settings and improves survival and quality of life when combined with 5-FU/LV. The success of these polychemotherapy regimens has greatly enhanced the clinical efficacy of 5-FU [6].

A key mechanism underlying 5-FU resistance in gastric cancer is the aberrant activation of DNA damage repair capabilities within the tumor cells [7]. 5-FU exerts its cytotoxic effects primarily by inhibiting thymidylate synthase (TS), thereby depleting the nucleotide pools necessary for DNA synthesis [8]. 5-FU is converted intracellularly into three main active metabolites: FdUMP, FdUTP, and FUTP. FdUMP inhibits thymidylate synthase (TS) by forming a stable ternary complex with TS and the folate cofactor, depleting dTTP and leading to DNA synthesis inhibition and thymineless cell death. FdUTP is incorporated into DNA, causing DNA damage and triggering repair mechanisms, while FUTP is incorporated into RNA, interfering with RNA processing and function and contributing to cytotoxicity [9]. Cancer cells, however, can evade this fate by enhancing various DNA repair pathways. The Base Excision Repair (BER) pathway is crucial for repairing base damage caused by 5-FU incorporation, and overexpression of its key components (e.g., APE1) is associated with resistance [10, 11]. Additionally, the Nucleotide Excision Repair (NER) and Mismatch Repair (MMR) systems are involved in handling DNA interstrand crosslinks and mismatches induced by 5-FU [12, 13]. Consequently, current research frontiers focus on combining DNA damage repair inhibitors with 5-FU. Strategies such as using PARP inhibitors to block the BER pathway or ATM/ATR inhibitors to disrupt DNA damage response signaling are being actively investigated [14, 15]. This synthetic lethality strategy aims to disable the DNA repair capacity of cancer cells, thereby resensitizing them to 5-FU and presenting a promising approach to overcome the clinical resistance. Looking ahead, personalized combination therapies tailored to the DNA repair profile of individual tumors could emerge as an effective strategy to reverse resistance and improve outcomes in gastric cancer.

The cell cycle-related and expression-elevated protein in tumor (CREPT/RPRD1B) is initially identified as an oncogene frequently overexpressed in multiple human cancers [16, 17]. RPRD1B is characterized as an RNA polymerase II (RNAPII)-interacting protein, primarily mediated by its conserved RNAPII C-terminal domain (CTD)-interacting domain (CID) [18, 19]. This interaction underpins its key role in regulating transcription. A pivotal mechanism involves RPRD1B facilitating the recruitment of RNAPII to gene promoters and simultaneously inhibiting its read-through past the poly(A) signal in termination regions, as exemplified by the CCND1 gene. It promotes a gene looping conformation that enables RNAPII recycling to the promoter post-termination and directly interacts with the RNAPII CTD. Beyond transcriptional regulation, RPRD1B drives cell proliferation and tumorigenesis by activating critical signaling pathways, most notably the Wnt/β-catenin and STAT pathways, which are central to cancer cell survival and growth [20–22].

In this study, we demonstrate that RPRD1B plays a crucial role in gastric cancer 5-FU chemoresistance by increasing DNA damage repair. Mechanistically, we found that RPRD1B enhanced the binding of RNA polymerase II to the promoter of the TOPBP1 gene, thereby activating its transcription. Notably, we demonstrated that patients with tumors that highly express RPRD1B have a dramatically weaker response to 5-FU drug-based chemotherapy. More importantly, we demonstrated that RPRD1B-targeting AAV overcame 5-FU resistance in gastric cancer. Our data collectively have identified a molecular mechanism underlying RPRD1B-mediated chemoresistance, indicating that RPRD1B may be a potential target for gastric cancer treatment.

## Methods

### Cell culture

All cell lines used in this study were maintained in a humidified incubator at 37 °C with 5% CO₂. The 5-FU-resistant gastric cancer cell lines AGS-5-FU-R and MGC803-5-FU-R were established by continuous, stepwise exposure of the parental AGS and MGC803 cells to progressively increasing concentrations of 5-FU.

### Cell counting Kit-8

Indicated cells were seeded in 96-well plates at a density of 2,000 cells per well. After the appropriate treatment period, cell viability was assessed using the Cell Counting Kit-8 (CCK-8). Briefly, 10 µL of CCK-8 reagent was mixed with 90 µL of serum-free medium, and the mixture was added to each well. Following a 1-hour incubation, absorbance was measured at 450 nm using a PowerWave XS microplate reader.

### Colony formation assay

Cells were seeded in the 6-well plates at a density of 1 × 10³ cells per well and continuously treated with 5-FU. Following 10 days of culture, cells were fixed and stained with crystal violet. Representative results from three independent experiments are shown.

### In vitro invasion and migration assays

Invasion and migration assays were performed using Transwell chambers (8 μm pore size). For the invasion assay, the upper surface of the membrane was coated with Matrigel; for the migration assay, no coating was used. Cells (1 × 10⁶) were resuspended in the 300 µL of serum-free medium and seeded into the upper chamber, while the lower chamber was filled with 200 µL of medium containing 10% FBS. After 24 h, cells on the lower surface were fixed with methanol, stained with 0.5% crystal violet, and counted under a light microscope (100× magnification) in three random fields. All experiments were performed in triplicate. Cell counts were obtained from captured images using ImageJ software, and statistical analysis was conducted with GraphPad Prism.

### Evaluation of apoptosis using Annexin V/propidium iodide staining

Cells were seeded in 60-mm dishes at a density of 3 × 10⁵ cells per dish and treated as indicated in the experimental design. Following the designated incubation period, cells were detached using trypsin-EDTA, washed with phosphate-buffered saline (PBS), and resuspended in 1× binding buffer. Apoptosis was then assessed by staining with Annexin V-FITC and PI-PE for 15 min in the dark, followed by analysis on a flow cytometer.

### Western blotting

Proteins were extracted using RIPA the lysis buffer supplemented with protease and phosphatase inhibitor cocktails. The following primary antibodies were used: anti-CREPT/RPRD1B (1:1,000; Thermo Fisher Scientific, cat. no. PA5-148238), anti-TOPBP1 (1:1,000; CST, cat. no. 14342), anti-p-γH2AX (1:1,000; CST, cat. no. 9718), and β-Actin (1:3,000; Proteintech, cat. no. 23660-1-AP). The corresponding secondary antibody was obtained from ABclonal Biotech Co., Ltd. Uncropped Gels and Blots images listed in the Supplementary Material. Detailed immunoblotting procedures were performed as previously described [22].

### Real-time quantitative PCR (QRT-PCR) analysis

Total RNA was extracted using TRIzol reagent (Invitrogen). Reverse transcription was performed with a Quantscript RT Kit (TIANGEN Biotech). Quantitative real-time PCR (qRT-PCR) was carried out using a Talent qPCR PreMix (SYBR Green) Kit (TIANGEN Biotech) on a Roche LightCycler instrument under the following cycling conditions: 95 °C for 5 s (denaturation), 60 °C for 10 s (annealing), and 72 °C for 15 s (extension). Primer sequences used are listed in the Supplementary Material, as detailed in Table S1.

### Chromatin immunoprecipitation (ChIP) assay

In brief, 1 × 10⁷ cells were fixed with the 1% formaldehyde, and chromatin was fragmented by sonication to lengths of 100–500 bp. Lysates were immunoprecipitated with the indicated antibodies for chromatin immunoprecipitation (ChIP). DNA was eluted and used as template for quantitative PCR (qPCR). Input controls were prepared from the supernatant collected prior to immunoprecipitation. The promoter region of interest was amplified by PCR using the primers listed in the Supplementary Material, as detailed in Table S2.

### Immunofluorescence staining

Cells were seeded onto coverslips in 6-well plates and incubated overnight at 37 °C. Cells were fixed in 4% paraformaldehyde for 20 min and permeabilized with 0.3% Triton X-100 for 10 min. After blocking with the 10% goat serum for 1 h at room temperature, cells were incubated overnight at 4 °C with the indicated primary antibodies, followed by incubation for 1 h with species-matched secondary antibodies conjugated to FITC (green) or TRITC (Jackson ImmunoResearch). Images were acquired using a confocal laser scanning microscope (Olympus FV10i-Oil), and protein co-localization was evaluated by merging the corresponding fluorescence channels.

### Immunohistochemistry staining and scoring

As described previously [21], formalin-fixed, paraffin-embedded (FFPE) tissue sections were processed for immunohistochemistry (IHC). Detailed staining procedures are provided in the Supplementary Materials. Protein expression was quantified using the H-score system, wherein staining intensity was independently evaluated by two experienced pathologists and scored as 0 (negative), 1 (weak), 2 (moderate), or 3 (strong). The H-score (range 0–300) was calculated for each sample as follows: (% of cells scored 1 × 1) + (% of cells scored 2 × 2) + (% of cells scored 3 × 3). Samples were then classified into high- and low-expression groups based on a cut-off set at the median H-score.

### In vivo tumor experiments

All animal experiments were approved by the ethical committee of Jiaxing University. To establish xenograft models, Cells were subcutaneously injected into the BALB/c nude mice.

### AAV construction and virus package

The lentiviral expression vector pZDonor_Seq1-U6-shRNA-hEF1a-EGFP-2 A-Puro was used to construct shRNA-expressing constructs. The target shRNA sequence against RPRD1B was 5′-GCACGAAGATTAGGTGCATTT-3′. The scrambled sequence was (5′-AAACGTGACACGTTCGGAGA.

ACGAA-3′) served as the negative control. For adeno-associated virus (AAV) production, an AAV2 packaging system was employed, which included the following components: the plasmid pRV1 (encoding the AAV2 Rep and Cap genes), the adenovirus helper plasmid pFΔ6, and the AAV proviral plasmids (AAV-shNC and AAV-shRPRD1B). These AAV plasmids contained the recombinant shRNA expression cassette flanked by AAV2 inverted terminal repeats (ITRs).

### Statistical analysis

All experiments were performed in triplicate. Data are expressed as mean ± SD. Differences between two groups were assessed using a two-tailed Student’s t-test.

### Data availability

Data are available from the corresponding author upon reasonable request.

## Results

### Elevated RPRD1B expression correlates with chemoresistance in gastric cancer

**Fig. 1:**
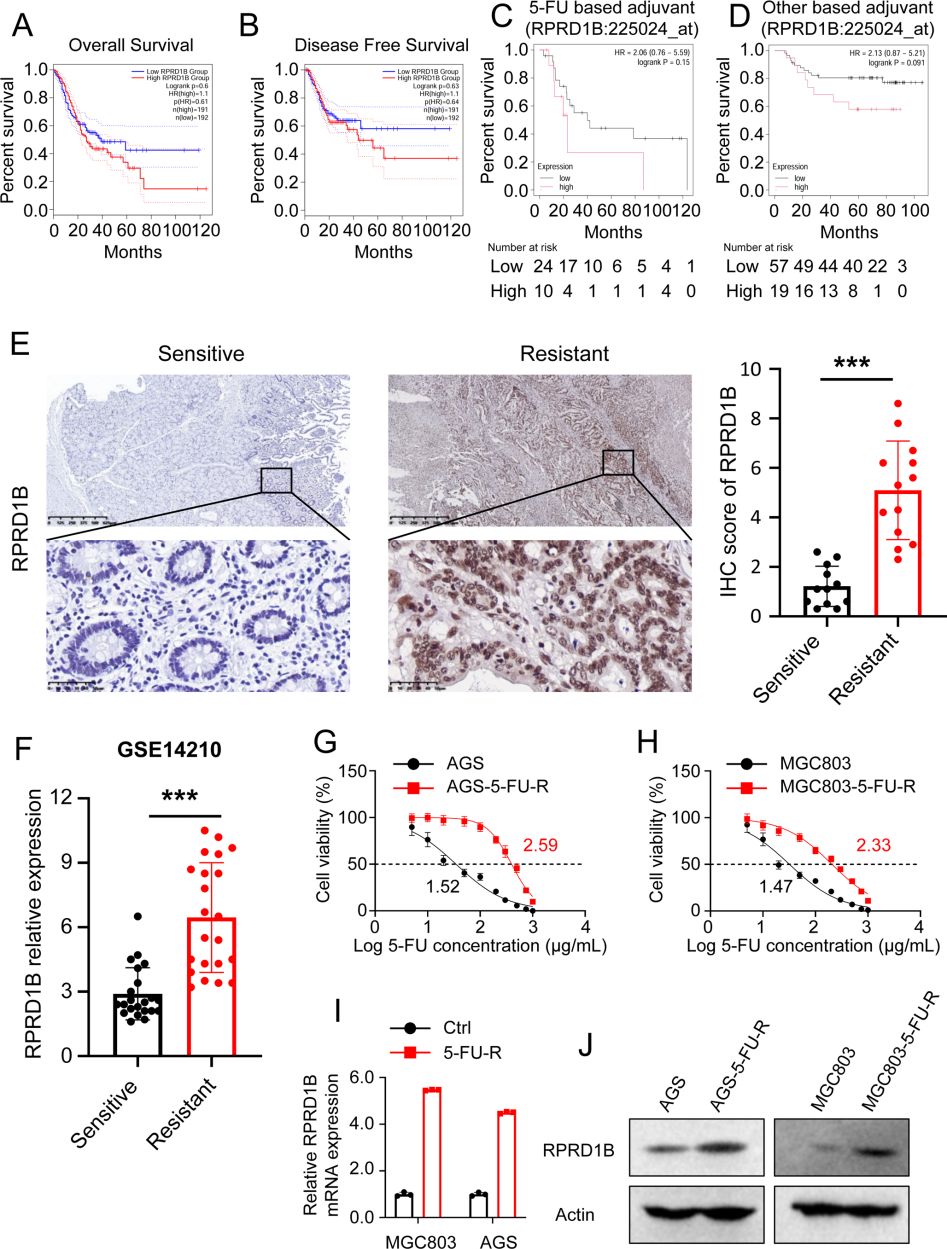
Elevated RPRD1B expression is clinically associated with chemoresistance in gastric cancer. (**A**) Overall survival analysis of gastric cancer patients stratified by RPRD1B expression level (GEPIA database). (**B**) Disease-free survival analysis of gastric cancer patients stratified by RPRD1B expression level (GEPIA database). (**C**) Overall survival analysis by KM-plotter showing the prognostic impact of RPRD1B expression in GC patients treated with 5-FU. (**D**) Overall survival analysis by KM-plotter showing the prognostic impact of RPRD1B expression in GC patients treated with other chemotherapeutic agents (e.g., oxaliplatin or paclitaxel). (**E**) Representative immunohistochemical images of RPRD1B expression in paired 5-FU-resistant and 5-FU-sensitive gastric tumor tissues (left). Quantitative analysis of RPRD1B staining intensity in patient samples is shown (right). (**F**) RPRD1B mRNA expression is significantly upregulated in 5-FU-resistant tumor tissues compared with sensitive counterparts. (**G-H**) Establishment of 5-FU-resistant AGS and MGC803 cell lines via stepwise exposure to increasing concentrations of 5-FU. (**I**) qRT-PCR analysis of RPRD1B mRNA levels in parental and 5-FU-resistant cell lines. (**J**) Western blot analysis of RPRD1B/RPRD1B protein levels in parental and 5-FU-resistant cell lines. Data were shown as the mean ± SD and analyzed using an unpaired two-tailed t-test. **: *P* < 0.01; ***: *P* < 0.001

To investigate the clinical role of RPRD1B in gastric cancer (GC), we used the public online database GEPIA2 to evaluate the prognostic value of CREPT/RPRD1B. Survival analysis revealed that high expression of CREPT/RPRD1B was significantly associated with poor prognosis in GC, correlating with both shorter overall survival and reduced disease-free survival (Fig. 1A and B). In the subgroup analysis, we utilized the KM plotter to evaluate the association between RPRD1B expression and the response to 5-FU chemotherapy in gastric cancer. The results revealed that among patients receiving chemotherapy, higher RPRD1B expression was correlated with a worse overall survival (Figs. 1C). Furthermore, we also observed that patients with high RPRD1B expression who received other chemotherapeutic agents (such as oxaliplatin or paclitaxel) also exhibited poor prognosis (Figs. 1D). These findings collectively suggest that RPRD1B may regulate drug sensitivity in gastric cancer.

To verify the possible role of RPRD1B in chemoresistance, we performed immunohistochemical staining for RPRD1B in 25 cases of gastric cancer treated with the 5-FU-based neoadjuvant chemotherapy. The subjects were divided into two groups based on their responsiveness to the treatment. The results demonstrated that the expression of RPRD1B in chemoresistant tumors was significantly higher than that in sensitive tumors (Fig. 1E). We analyzed the gene expression profiles from 22 patients who initially responded to 5-FU but were re-biopsied after they developed resistance to 5-FU. Our analysis demonstrated that RPRD1B expression was significantly elevated in the non-responder group compared to the responder group (Fig. 1F).

To investigate the biological function of RPRD1B in 5-FU-resistant cells, we established resistant cell lines through long-term, low-dose, and stepwise induction, ultimately obtaining two lines capable of stable proliferation under the high 5-FU concentrations (Fig. 1G and H). We found that RPRD1B expression, at both the mRNA and protein levels, was significantly higher in the 5-FU-resistant cells than in the wild-type AGS and MGC803 cells (Fig. 1I and J). Collectively, these results suggest that RPRD1B may be involved in regulating gastric cancer cell sensitivity to the chemotherapy drug 5-FU.

### RPRD1B knockdown sensitizes gastric cancer cells to 5-FU treatment in vitro

**Fig. 2:**
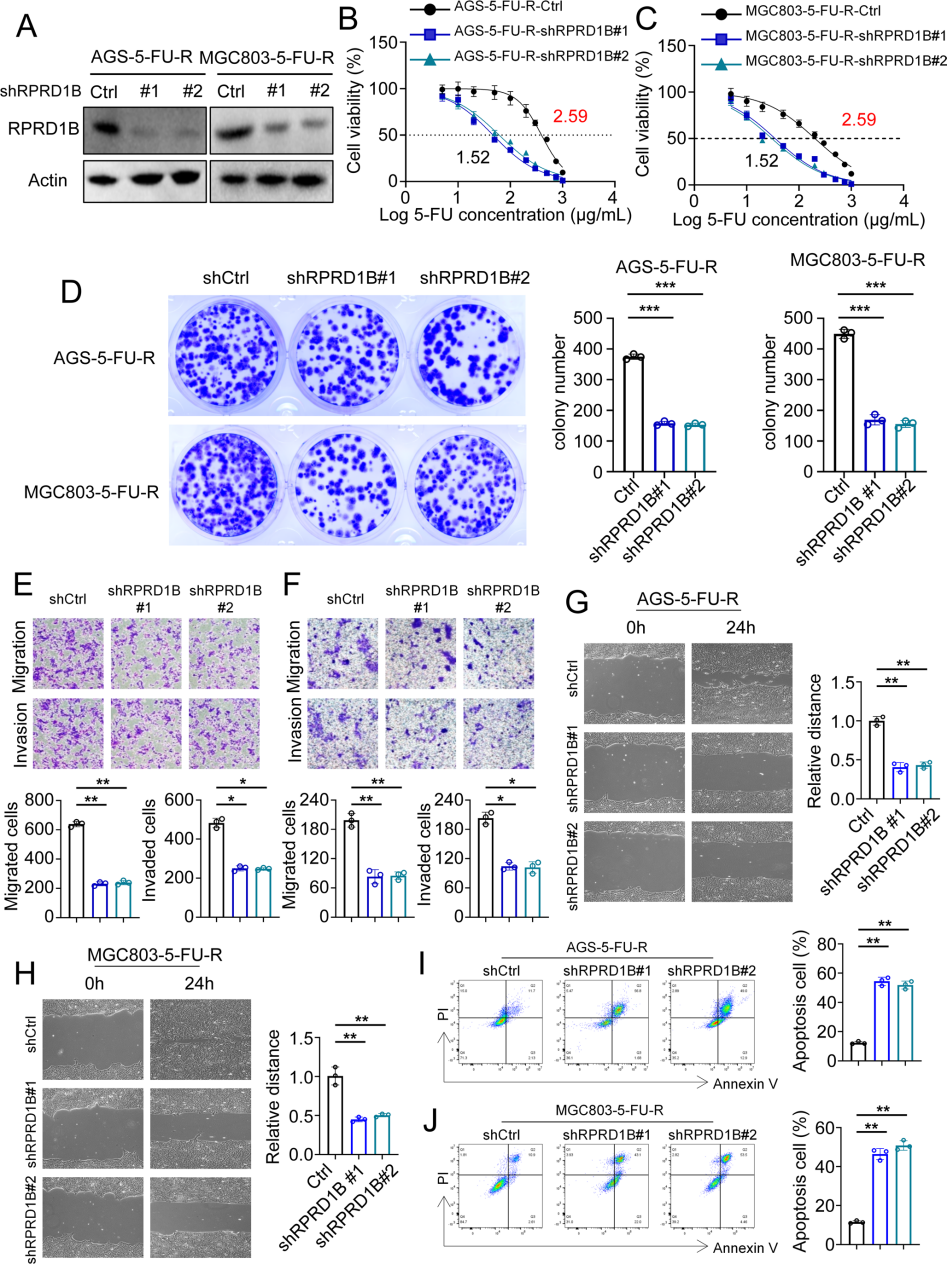
RPRD1B knockdown renders 5-FU-resistant gastric cancer cells more sensitive to 5-FU treatment in vitro. (**A**) Western blot were conducted to detect the expression levels of RPRD1B in the control and RPRD1B-knockdown AGS-5-FU-R and MGC803-5-FU-R cells. (**B-C**) The IC50 of 5-FU was measured in RPRD1B-knockdown AGS-5-FU-R and MGC803-5-FU-R cells. (**D**) Colony formation assays were performed to examine the proliferation of the control and RPRD1B-knockdown AGS-5-FU-R and MGC803-5-FU-R cells. (**E-F**) Transwell migration and matrigel invasion assays were performed to examine the migration and invasion of the control and RPRD1B-knockdown AGS-5-FU-R and MGC803-5-FU-R cells. (**G-H**) wound healing assays were performed to examine the migration of the control and RPRD1B-knockdown AGS-5-FU-R and MGC803-5-FU-R cells. (**I-J**) Apoptosis assay were performed to examine the migration of the control and RPRD1B-knockdown AGS-5-FU-R and MGC803-5-FU-R cells. Data were shown as the mean ± SD and analyzed using an unpaired two-tailed t-test. **: *P* < 0.01; ***: *P* < 0.001

To investigate the role of RPRD1B in 5-FU resistance, we stably knocked down RPRD1B using short hairpin RNAs (shRNAs) in AGS-5-FU-R and MGC803-5-FU-R cells and confirmed a notable decrease in RPRD1B expression at both the protein and mRNA levels (Fig. 2A and Figure S1A). We first examined the effect of RPRD1B knockdown on the GC cell resistance to 5-FU. The results showed that depletion of RPRD1B significantly enhanced 5-FU sensitivity in both AGS-5-FU-R and MGC803-5-FU-R cells (Fig. 2B and C). Colony formation assays further revealed that the clonogenic ability of these 5-FU-resistant GC cells was dramatically inhibited after RPRD1B knockdown (Fig. 2D), indicating that RPRD1B knockdown significantly impaired cell proliferation. Functional assays further revealed that RPRD1B knockdown suppressed cell migration and invasion, as demonstrated by Transwell migration, Matrigel invasion, and wound healing assays (Fig. 2E and H). Additionally, flow cytometry analysis showed that RPRD1B knockdown significantly increased apoptosis in response to 5-FU treatment (Fig. 2I and J), suggesting that RPRD1B may confer 5-FU resistance, at least in part, by suppressing apoptosis in gastric cancer cells. At last, we conducted RPRD1B rescue experiments in RPRD1B-knockdown cell lines. The results demonstrated that exogenous expression of RPRD1B reversed the phenotypes induced by RPRD1B knockdown, including restored cell viability, increased colony formation, and decreased reduced apoptosis under 5-FU treatment (Figure S2A-2E). Collectively, these results suggest that inhibition of RPRD1B may represent a promising therapeutic strategy for 5-FU-resistant gastric cancer.

### Overexpression of RPRD1B confers resistance to 5-FU in gastric cancer cells

**Fig. 3:**
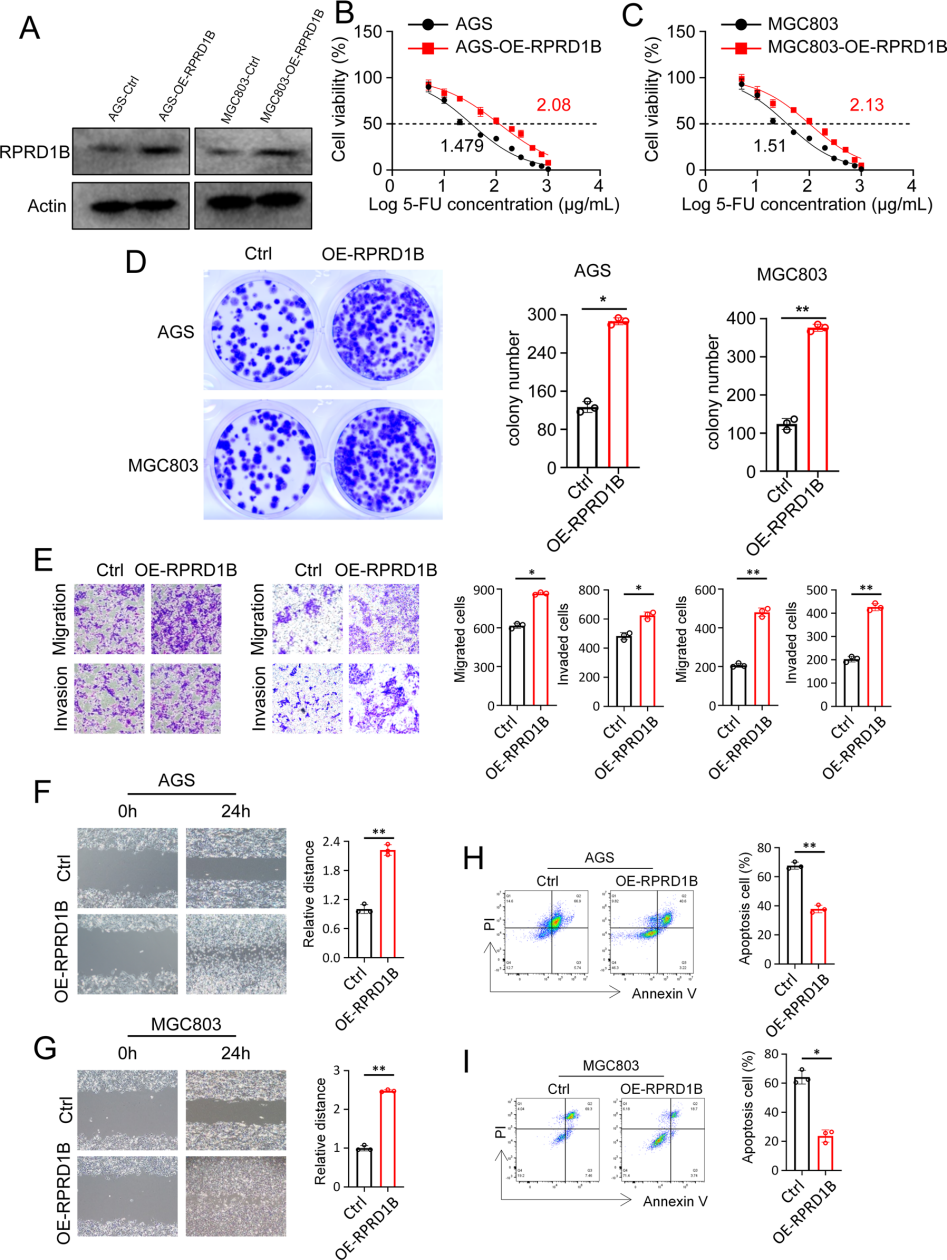
Ectopic expression of RPRD1B reduces 5-FU sensitivity in gastric cancer cells. (**A**) Western blot analysis of RPRD1B protein levels in control and RPRD1B-overexpressing AGS and MGC803 cells. (**B-C**) The IC50 of 5-FU was measured in the control and RPRD1B-Overexpression AGS and MGC803 cells under the 5-FU treatment. (**D**) Colony formation assays comparing proliferation of control and RPRD1B-overexpressing cells in the presence of 5-FU. (**E**) Transwell migration and matrigel invasion assays evaluating migratory and invasive capacities of control and RPRD1B-overexpressing cells under 5-FU treatment. (**F-G**) Wound-healing assays assessing cell migration of control and RPRD1B-overexpressing cells after 5-FU exposure. (**H-I**) Apoptosis assays measuring cell death in control and RPRD1B-overexpressing cells following 5-FU treatment. Data were shown as the mean ± SD and analyzed using an unpaired two-tailed t-test. **: *P* < 0.01; ***: *P* < 0.001

To further validate the above findings, we next examined whether overexpression of RPRD1B in parental gastric cancer cells would reduce their sensitivity to 5-FU. First, we stably transfected AGS and MGC803 cells with a RPRD1B-expression plasmid and confirmed a notable increase in RPRD1B levels by the western blot and qPCR (Fig. 3A and Figure S1B), We then measured the viability of these cells in the presence of 5-FU. As shown in Fig. 3B and C, ectopic overexpression of RPRD1B significantly enhanced cell viability under the 5-FU treatment (Fig. 3B and C). Colony formation assays further demonstrated that overexpression of RPRD1B enhanced the resistance of AGS and MGC803 cells to 5-FU (Fig. 3D). Transwell, Matrigel, and wound healing assays showed that RPRD1B overexpression enhanced cell migration and invasion upon 5-FU exposure. (Figure 3E and G). Moreover, we observed that overexpression of RPRD1B reduced the cell apoptosis induced by 5-FU treatment in AGS and MGC803 cells (Fig. 3H and I). Taken together, these data indicate that RPRD1B plays a critical role in conferring 5-FU resistance in gastric cancer cells.

### RPRD1B reduces DNA damage induced by 5-FU in GC cells

**Fig. 4:**
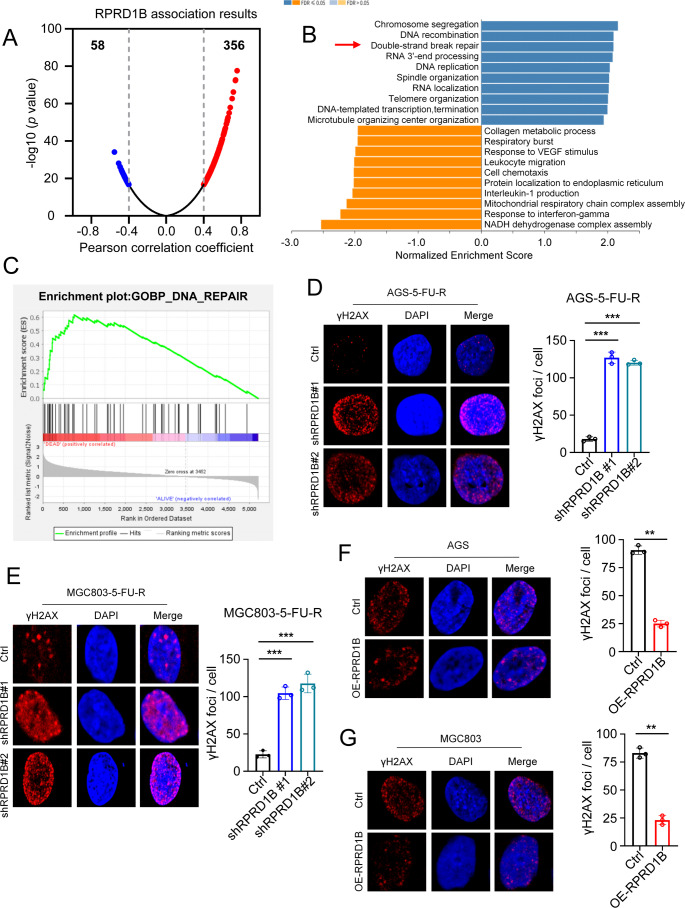
The expression of RPRD1B confers a protective effect against 5-FU-mediated DNA damage in gastric cancer cells. (**A**) The correlated genes identified by Pearson’s correlation coefficient in the GC cohort. (**B**) Significantly enriched GO annotations and KEGG pathways of RPRD1B in GC cohort. (**D-E**) Enrichment of genes in the GO DNA repair by GSEA. (E-F) Left: γ-H2AX staining (red) and DAPI staining (blue) in the control and RPRD1B-knockdown AGS-5-FU-R and MGC803-5-FU-R cells; Right: Quantification of mean γ-H2AX foci per cell. (**F-G**) Left: γ-H2AX staining (red) and DAPI staining (blue) in the control and RPRD1B-Overexpression AGS and MGC803 cells; Right: Quantification of mean γ-H2AX foci per cell. Data were shown as the mean ± SD and analyzed using an unpaired two-tailed t-test. **: *P* < 0.01; ***: *P* < 0.001

To elucidate the mechanism by which RPRD1B induces chemoresistance, we first investigated its biological function in gastric cancer. A co-expression network for RPRD1B was constructed using the LinkedOmics database [23], which identified 356 positively correlated and 58 negatively correlated genes (Fig. 4A). Gene Ontology (GO) analysis revealed that genes co-expressed with RPRD1B were significantly enriched in biological processes related to chromosome segregation, DNA recombination, double-strand break repair, and RNA 3’-end processing (Fig. 4B). Gene set enrichment analysis (GSEA) of the 356 positively correlated genes further confirmed strong enrichment of the DNA damage repair hallmark pathway (Fig. 4C). Given that enhanced DNA damage repair is a key mechanism of chemotherapy resistance, we hypothesized that RPRD1B promotes 5-FU resistance in gastric cancer through this pathway.

To examine the role of RPRD1B in DNA damage repair following 5-FU treatment, we assessed its effect on γ-H2AX levels, a well-established marker of DNA double-strand breaks. Immunofluorescence imaging revealed that RPRD1B knockdown in 5-FU-resistant cell lines (AGS-5-FU-R and MGC803-5-FU-R) significantly increased the formation of γ-H2AX nuclear foci (Fig. 4D and E). we also conducted RPRD1B rescue experiments in AGS-5-FU-R-shRPRD1B and MGC803-5-FU-R-shRPRD1B cells. The result demonstrated that exogenous expression of RPRD1B reversed the decreased γH2AX foci accumulation under 5-FU treatment (Figure S3A-3B). In contrast, overexpression of RPRD1B in parental AGS and MGC803 cells markedly reduced γ-H2AX foci accumulation after 5-FU exposure (Fig. 4F and G). These results suggest that RPRD1B plays an important role in promoting the repair of DNA double-strand breaks induced by chemotherapy.

### RPRD1B promotes DNA damage repair by facilitating the transcription of TOPBP1 expression

**Fig. 5:**
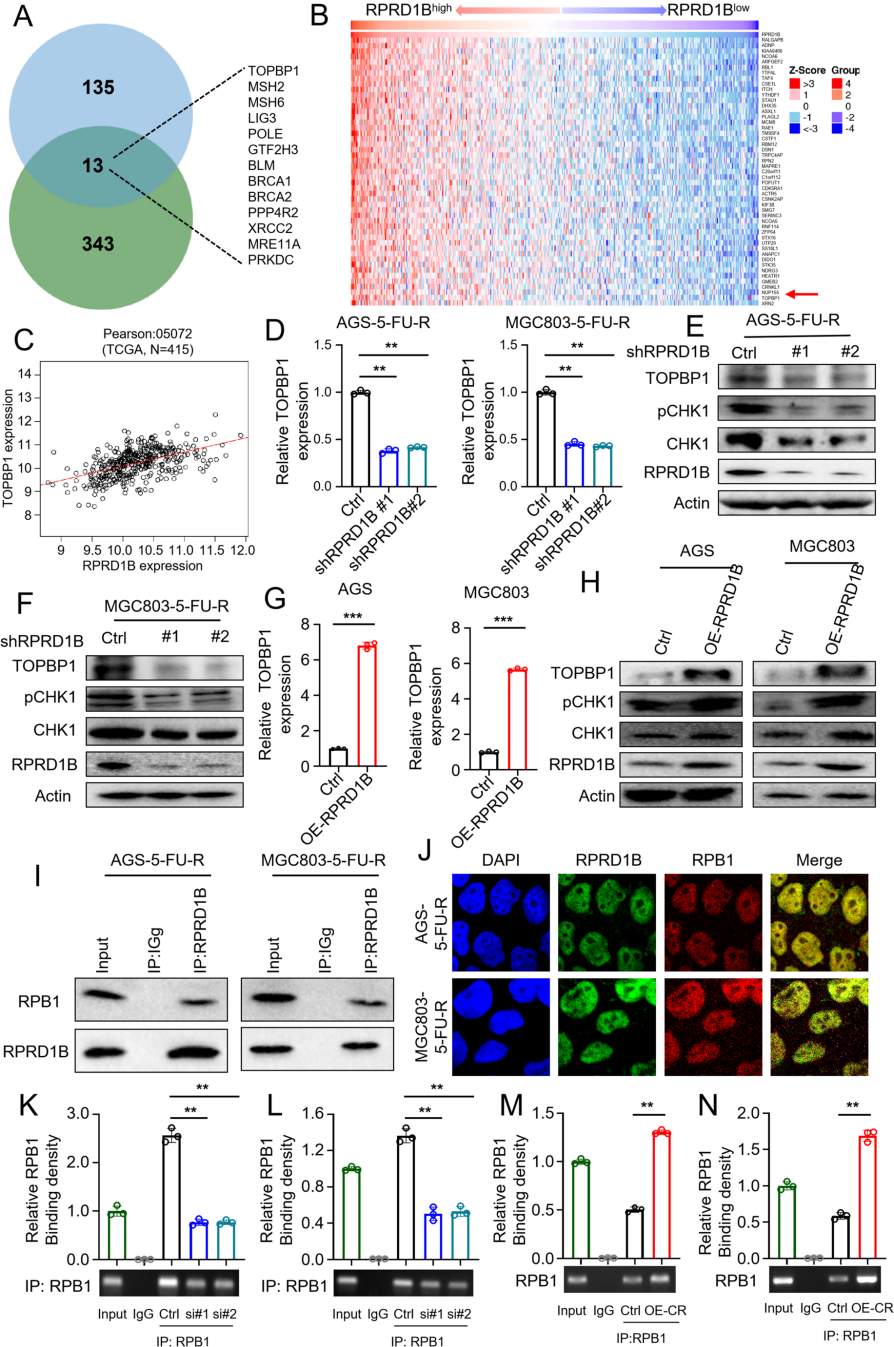
RPRD1B/RPRD1B transcriptionally upregulates TOPBP1 by recruiting RNAPII. (**A**) Venn diagram analysis identifies DNA damage repair (DDR)-related target genes regulated by RPRD1B. (**B**) Heatmap of the top 50 genes positively (red) and negatively (green) correlated with RPRD1B expression in gastric cancer (GC). (**C**) Spearman correlation analysis (LinkedOmics) of RPRD1B and TOPBP1 mRNA expression in GC tissues. (**D**) RT-qPCR analysis of TOPBP1 mRNA levels in control vs. RPRD1B-knockdown AGS-5-FU-R and MGC803-5-FU-R cells. (**E-F**) Western blot analysis of TOPBP1 protein levels and CHK1 phosphorylation in control vs. RPRD1B-knockdown AGS-5-FU-R and MGC803-5-FU-R cells. (**G**) RT-qPCR analysis of TOPBP1 mRNA levels in control vs. RPRD1B-overexpressing AGS and MGC803 cells treated with 5-FU. (**H**) Western blot analysis of TOPBP1 protein levels and CHK1 phosphorylation in control vs. RPRD1B-overexpressing AGS and MGC803 cells treated with 5-FU. (**I**) Immunoprecipitation (IP) assays detect interaction between RPRD1B and RPB1 in AGS-5-FU-R and MGC803-5-FU-R cells. (**J**) Immunofluorescence staining showing nuclear co-localization of RPRD1B and RPB1. (**K-L**) ChIP-qPCR analysis of RPB1 binding at the TOPBP1 promoter in control vs. RPRD1B-knockdown AGS-5-FU-R and MGC803-5-FU-R cells. (**M-N**) ChIP-qPCR analysis of RPB1 binding at the TOPBP1 promoter in control vs. RPRD1B-overexpressing AGS and MGC803 cells treated with 5-FU. Data were shown as the mean ± SD and analyzed using an unpaired two-tailed t-test. **: *P* < 0.01; ***: *P* < 0.001

To investigate how RPRD1B regulates DNA damage repair in 5-FU-resistant gastric cancer cells, we analyzed DDR-related genes that were positively correlated with RPRD1B expression. This analysis identified 13 DDR-related genes that are potentially regulated by RPRD1B (Fig. 5A). We focused on the TOPBP1 gene primarily because it showed the highest correlation with RPRD1B expression among all DDR-related genes (Fig. 5B). Correlation analysis of the TCGA dataset further confirmed a strong positive association between RPRD1B and TOPBP1 mRNA levels in gastric cancer tissues (Fig. 5C). We next examined whether TOPBP1 is directly regulated by RPRD1B. Results showed that RPRD1B knockdown significantly reduced TOPBP1 expression at both the protein and mRNA levels in AGS-5-FU-R and MGC803-5-FU-R cells (Fig. 5D and F). In addition, we found that the phosphorylation level of CHK1, a key component downstream of TOPBP1 [24], was also reduced. Conversely, ectopic overexpression of RPRD1B markedly increased TOPBP1 expression and the phosphorylation level of CHK1 in parental gastric cancer cells (Fig. 5G and H). To investigate the functional role of RPRD1B binding to RNA polymerase II (RNAPII) in regulating TOPBP1 transcription, we performed immunoprecipitation (IP) experiments in AGS-5-FU-R and MGC803-5-FU-R cells. The results confirmed a direct interaction between RPRD1B and RPB1, the largest subunit of RNAPII (Fig. 5I). Immunofluorescence staining further confirmed the co-localization of RPRD1B and RPB1 within the nucleus(Fig. 5J). To determine whether RPRD1B influences the occupancy of RNAPII at the TOPBP1 promoter, we conducted chromatin immunoprecipitation followed by PCR (ChIP-PCR). The results showed that knockdown of RPRD1B significantly reduced the enrichment of both RNAPII and RPRD1B at the TOPBP1 promoter region (Fig. 5K and L and Figure S4A-S4B). Conversely, overexpression of RPRD1B markedly increased the occupancy of both factors at the same locus (Fig. 5M and N and Figure S4C-S4D). Collectively, these findings demonstrate that RPRD1B promotes TOPBP1 expression in gastric cancer cells by facilitating the recruitment of RNAPII at its promoter, suggesting a transcriptional mechanism through which RPRD1B may enhance DNA damage repair in response to the 5-FU chemotherapy.

### TOPBP1 is required for RPRD1B-mediated chemoresistance

**Fig. 6:**
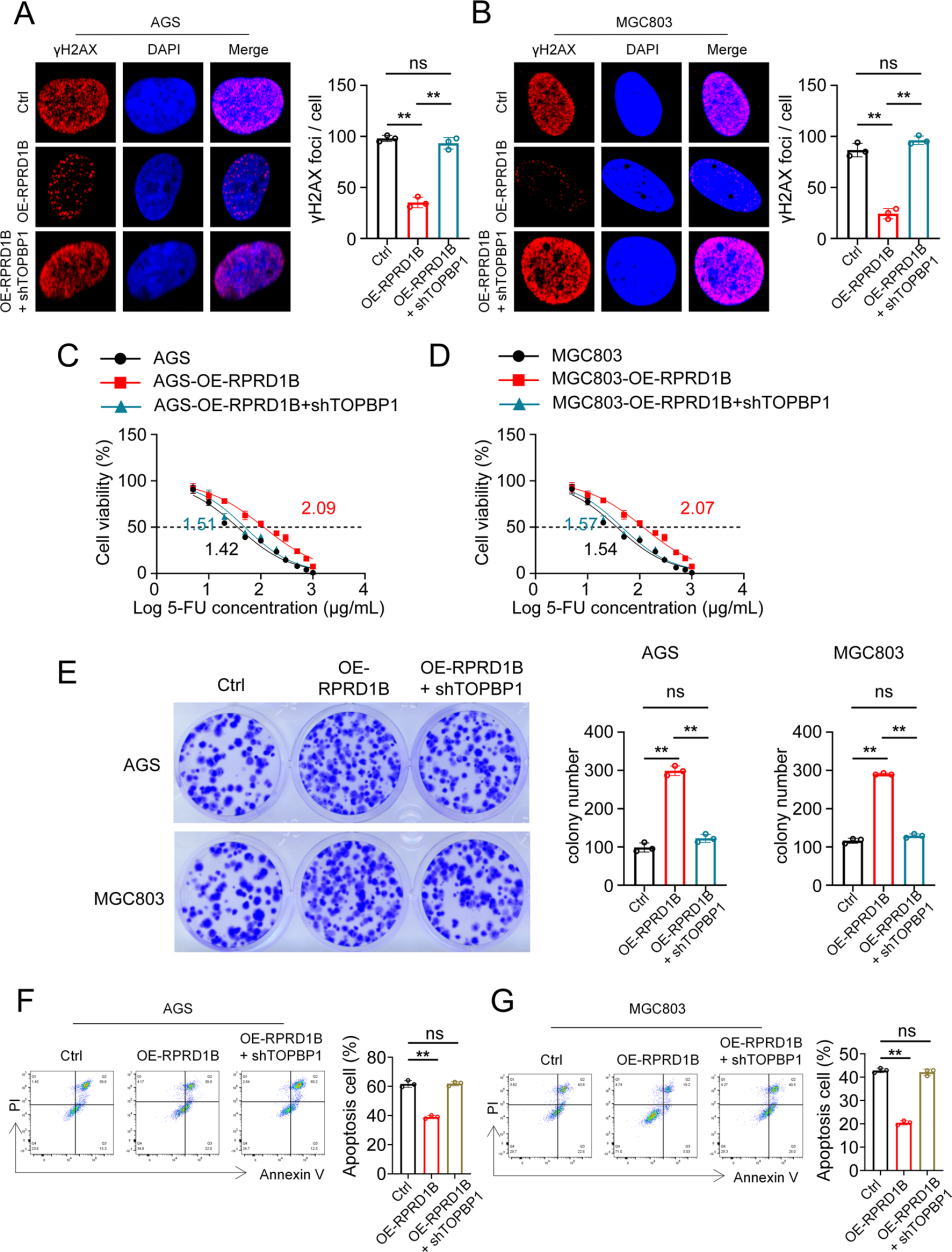
TOPBP1 is essential for RPRD1B-mediated chemoresistance. (**A-B**) Left: γ-H2AX staining (red) and DAPI staining (blue) in the control or TOPBP1-targeted shRNA into RPRD1B-overexpressing AGS and MGC803 cells, followed by 5-FU treatment; Right: Quantification of mean γ-H2AX foci per cell. (**C-D**) The IC50 of 5-FU was measured in the control or TOPBP1-targeted shRNA into RPRD1B-overexpressing AGS and MGC803 cells, followed by 5-FU treatment. (**E**) Colony formation assays comparing proliferation of control or TOPBP1-targeted shRNA into RPRD1B-overexpressing AGS and MGC803 cells in the presence of 5-FU.(**F-G**) Apoptosis assays measuring cell death in the control or TOPBP1-targeted shRNA into RPRD1B-overexpressing AGS and MGC803 cells, followed by 5-FU treatment. Data were shown as the mean ± SD and analyzed using an unpaired two-tailed t-test. **: *P* < 0.01; ***: *P* < 0.001

To determine the role of TOPBP1 in RPRD1B-mediated chemoresistance, we first examined whether TOPBP1 is required for the DNA damage repair pathway promoted by RPRD1B. The results showed that overexpression of RPRD1B increased the formation of γ-H2AX nuclear foci after 5-FU exposure, whereas this effect was diminished upon silencing TOPBP1, indicating that TOPBP1 is essential for RPRD1B-mediated DNA damage repair under the 5-FU drug treatment (Fig. 6A and B). Next, we tested whether the RPRD1B/TOPBP1 axis functionally confers resistance to 5-FU-induced growth inhibition in gastric cancer cells. We introduced control or TOPBP1-targeted shRNA into RPRD1B-overexpressing AGS and MGC803 cells, followed by the 5-FU treatment. Cell viability assays revealed that ectopic expression of RPRD1B enhanced cell growth in the presence of 5-FU, an effect that was attenuated by TOPBP1 knockdown (Fig. 6C and D). Similar results were obtained in colony formation assays (Fig. 6E). We further investigated the effect of TOPBP1 on RPRD1B-mediated apoptosis in response to 5-FU treatment. Results showed that depletion of TOPBP1 in RPRD1B-overexpressing cells reversed the RPRD1B-mediated suppression of 5-FU-induced apoptosis in both AGS and MGC803 cell lines (Fig. 6F and G), suggesting that RPRD1B regulates DNA damage-associated apoptosis primarily through TOPBP1. In addition, we conducted TOPBP1 rescue experiments in AGS-5-FU-R and MGC803-5-FU-R cells. The results revealed that exogenous expression of TOPBP1 significantly reversed the phenotypes induced by RPRD1B knockdown, including restored cell viability, increased colony formation, reduced apoptosis, and decreased γH2AX foci accumulation under 5-FU treatment (Figure S5A-5G). In summary, these findings reveal an important mechanism by which RPRD1B modulates gastric cancer sensitivity to 5-FU in a TOPBP1-dependent manner.

### Targeting RPRD1B is a promising strategy for the therapy of 5-FU chemoresistance

**Fig. 7:**
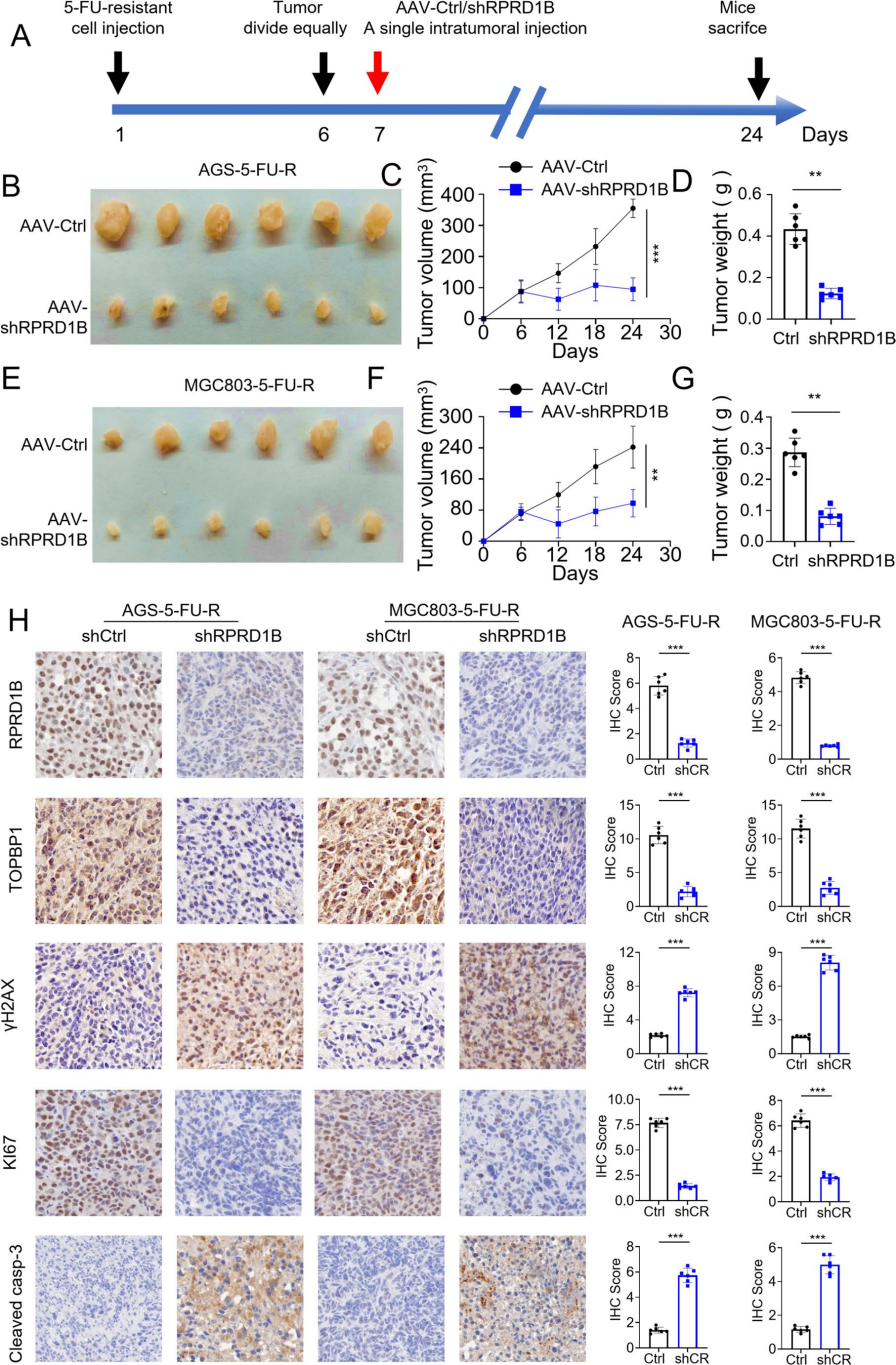
Targeting RPRD1B represents a promising therapeutic strategy for overcoming 5-FU chemoresistance. (**A**) Schematic of the experimental design. BALB/c nude mice (*n* = 6 per group) were subcutaneously inoculated with5 × 10^5^ 5-FU-resistant gastric cancer cells. When tumors reached a specified volume, AAV-shRPRD1B or control AAV was administered via intratumoral injection. (**B**,** E**) Representative tumor photographs from each group at the endpoint. (**C**,** F**) Tumor growth curves of the indicated xenografts. (**D**,** G**) Final tumor weights under each treatment condition.(**H**) Left: Representative IHC staining of RPRD1B, TOPBP1, γ-H2AX, Ki-67, and cleaved caspase-3 in tumors from the indicated groups. Right: Quantitative analysis of staining intensity for each marker. Data were shown as the mean ± SD and analyzed using an unpaired two-tailed t-test. **: *P* < 0.01; ***: *P* < 0.001

To explore potential clinical applications, we developed an AAV-based system to deplete RPRD1B expression in 5-FU-resistant cell lines (AGS-5-FU-R and MGC803-5-FU-R). The efficiency of the AAV-delivered shRNA targeting RPRD1B (AAV-shRPRD1B) was confirmed by GFP immunofluorescence (Figure S6). To determine whether AAV-shRPRD1B exhibits therapeutic efficacy against 5-FU-resistant tumors, we first allowed tumors to establish in the mice prior to initiating AAV-based therapy (Fig. 7A). In the AGS-5-FU-R mouse model, treatment with AAV-shRPRD1B significantly suppressed tumor growth, as reflected by reduced tumor volume (Fig. 7B and D). A similar inhibitory effect was observed in the MGC803-5-FU-R model (Fig. 7E and G), indicating that targeting RPRD1B with AAV-shRPRD1B is an effective and robust strategy for treating the 5-FU-resistant tumors. Immunohistochemical staining and corresponding quantitative analysis further confirmed that, compared with the AAV-Ctrl group, AAV-shRPRD1B treatment reduced the expression of RPRD1B and its target gene TOPBP1 in tumor tissues (Fig. 7H). This reduction was accompanied by a concurrent upregulation of γ-H2AX (Fig. 7H), indicating effective suppression of the RPRD1B-mediated DNA damage repair pathway. These results demonstrate that AAV-shRPRD1B is an effective means of inhibiting RPRD1B expression in vivo. Furthermore, we observed a decrease in Ki67 (a proliferation marker) and an increase in cleaved caspase-8 (an apoptosis marker) (Fig. 7H), suggesting that RPRD1B knockdown not only impedes DNA damage repair but also suppresses tumor cell proliferation and promotes apoptosis. Collectively, these data demonstrate that targeting RPRD1B with AAV-delivered shRNA as a potent strategy for suppressing 5-FU-resistant tumors.

## Discussion

**Fig. 8:**
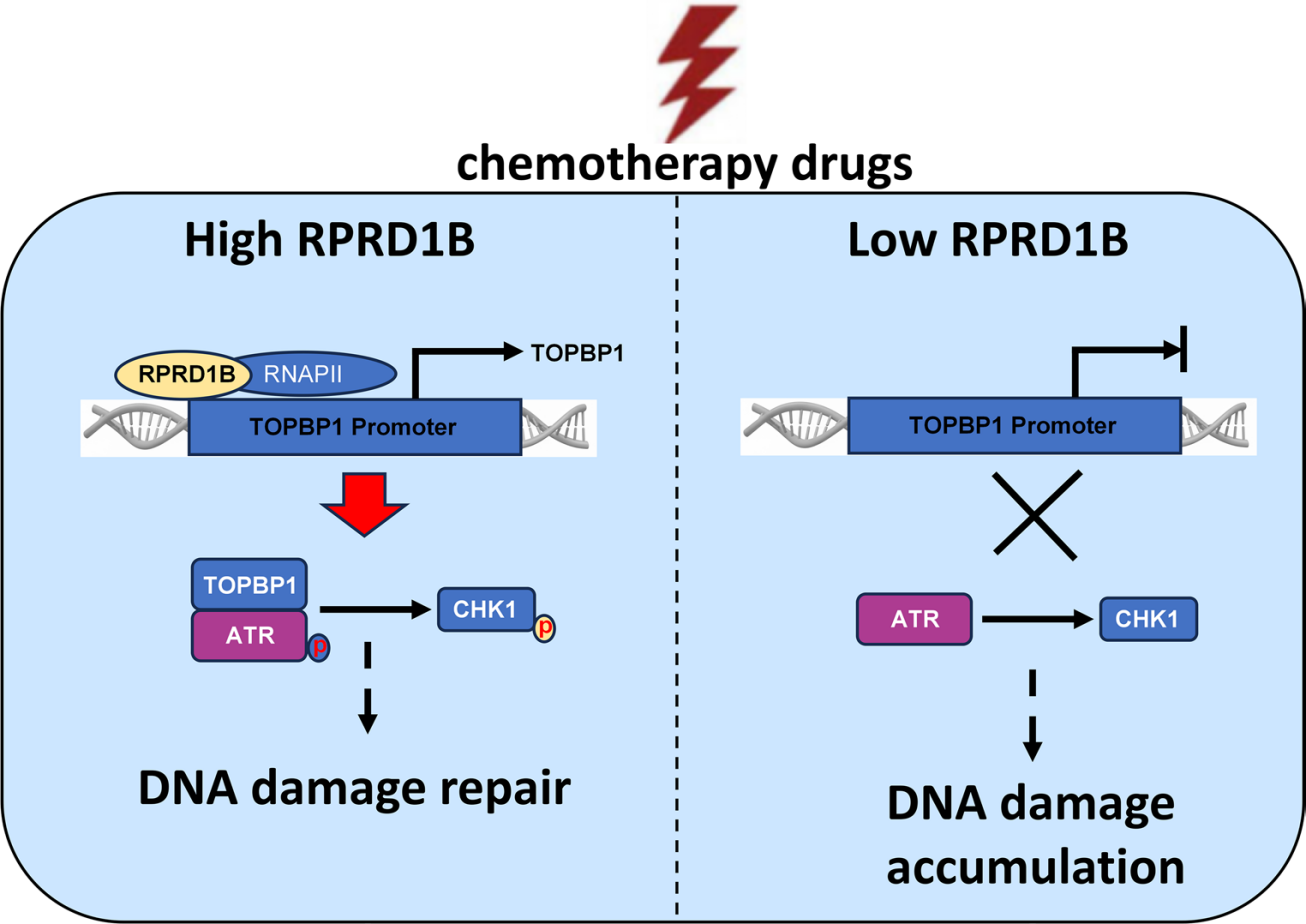
The schematic diagram depicts the proposed mechanism by which RPRD1B promotes chemoresistance in gastric cancer cells. Specifically, RPRD1B recruits RNA polymerase II to the TOPBP1 promoter, leading to transcriptional upregulation of TOPBP1. This in turn activates the ATR-Chk1 DNA damage repair pathway, ultimately conferring resistance to chemotherapy

The global incidence of gastric cancer has been steadily increasing in recent years, posing a significant public health burden. A central challenge in its clinical management is the emergence and evolution of chemoresistance. Although multiple chemotherapeutic regimens—such as combinations based on platinum agents, taxanes, and fluoropyrimidines—are used to treat the disease, tumor cells can develop resistance through various adaptive mechanisms [25–27]. Among the chemotherapeutic agents available, 5-fluorouracil (5-FU) serves as a backbone drug and is widely used in both adjuvant and neoadjuvant settings for gastric cancer. However, its long-term effectiveness is often severely limited by the development of resistance. This study focuses on RPRD1B (also known as RPRD1B), the human homolog of the yeast transcription termination factor RTT103, which plays critical roles in transcription. RPRD1B is overexpressed in multiple cancers, where its expression correlates with poor prognosis. Although we previously reported that RPRD1B exerts a dual oncogenic function in gastric cancer—promoting tumor progression by regulating fatty acid metabolism and interacting with Aurora B to accelerate cell cycle progression—its specific role in mediating 5-FU chemoresistance in this malignancy remains poorly understood [28, 29]. Our results suggested that knockdown of RPRD1B induces apoptosis in 5-FU-resistant GC cells and enhances their sensitivity to the 5-FU drug. Another important finding is that AAV-mediated knockdown of RPRD1B exhibits potent efficacy against the 5-FU-resistant tumors (Fig. 8).

Research has identified key mechanisms of 5-FU resistance, including thymidylate synthase (TYMS) overexpression, altered dihydropyrimidine dehydrogenase (DPD) activity, and dysregulation of pathways such as Wnt/β-catenin and PI3K/Akt [30–33]. However, a significant translational gap persists between this mechanistic knowledge and the development of clinically effective strategies to overcome resistance. Clarifying the precise regulatory networks of chemoresistance and developing targeted therapies or rational drug combinations are therefore critically needed to improve outcomes for gastric cancer patients. In this study, our data indicate that RPRD1B transcriptionally regulates TOPBP1 by recruiting RNAPII to its promoter in gastric cancer cells, proposing a mechanism by which it facilitates DNA repair and 5-FU chemoresistance. TOPBP1 is a checkpoint activator protein that employs its multiple BRCA1 carboxyl-terminal (BRCT) motifs as scaffolds to regulate diverse aspects of DNA metabolism, such as DNA damage checkpoint activation, replication, and transcription. Functioning as a critical mediator of the ATR-Chk1 pathway, TOPBP1 potentiates ATR kinase activity, an essential signal amplification step for an effective DNA damage response [24]. Positioned at a convergent point of multiple oncogenic pathways, TOPBP1 has become an attractive target for cancer therapy. Our results showed that RPRD1B transcriptionally upregulates TOPBP1 by recruiting RNA polymerase II to its promoter, thereby activating the ATR-Chk1 DNA damage repair pathway and conferring chemoresistance in gastric cancer cells. Therefore, targeting RPRD1B may also represent a therapeutic strategy for a broad spectrum of tumors characterized by high TOPBP1 expression.

Previous studies have reported that depletion of RPRD1B leads to the accumulation of persistent RNA: DNA hybrids (R-loops) during transcription, which can cause deleterious DNA double-strand breaks (DSBs), likely due to replication fork collision [34]. The loss of RPRD1B not only elevates persistent R-loops and DSBs but also impairs their repair, as RPRD1B simultaneously functions to stabilize Artemis. Further investigations revealed that RPRD1B depletion induces a state of heightened genomic instability, in which caspase-dependent degradation of the MLH1-PMS2 heterodimer generates an indirect DNA mismatch repair–deficient phenotype [35]. To further explore the mechanisms underlying drug resistance in gastric cancer, we integrated bioinformatic and cellular experimental approaches. Our results demonstrated marked activation of the DNA repair signaling pathway in the 5-FU-resistant gastric cancer cells. Importantly, knockdown of TOPBP1—a key mediator of this pathway—effectively reversed 5-FU resistance, supporting the functional relevance of DNA repair in chemoresistance. Further mechanistic studies indicated that RPRD1B promotes 5-FU resistance by modulating this same DNA repair pathway. Although the role of RPRD1B in DNA repair remains underexplored, our findings highlight its significance and suggest its potential as a therapeutic target for overcoming chemotherapy resistance in gastric cancer.

## Electronic Supplementary Material

Below is the link to the electronic supplementary material.

Supplementary Material 1

Supplementary Material 2

## Data Availability

Data are available from the corresponding author upon reasonable request.
