# Supplementary figures and images for: Targeting RPRD1B overcomes chemoresistance in gastric cancer by suppressing the TOPBP1-mediated DNA damage repair pathway
Source: Cell Oncol (Dordr). 2026 Aug 2;49(3):84. doi: 10.1007/s13402-026-01227-0 (PMC13428727; doi:10.1007/s13402-026-01227-0)

**Gels and Blots image**

**Western blotting**


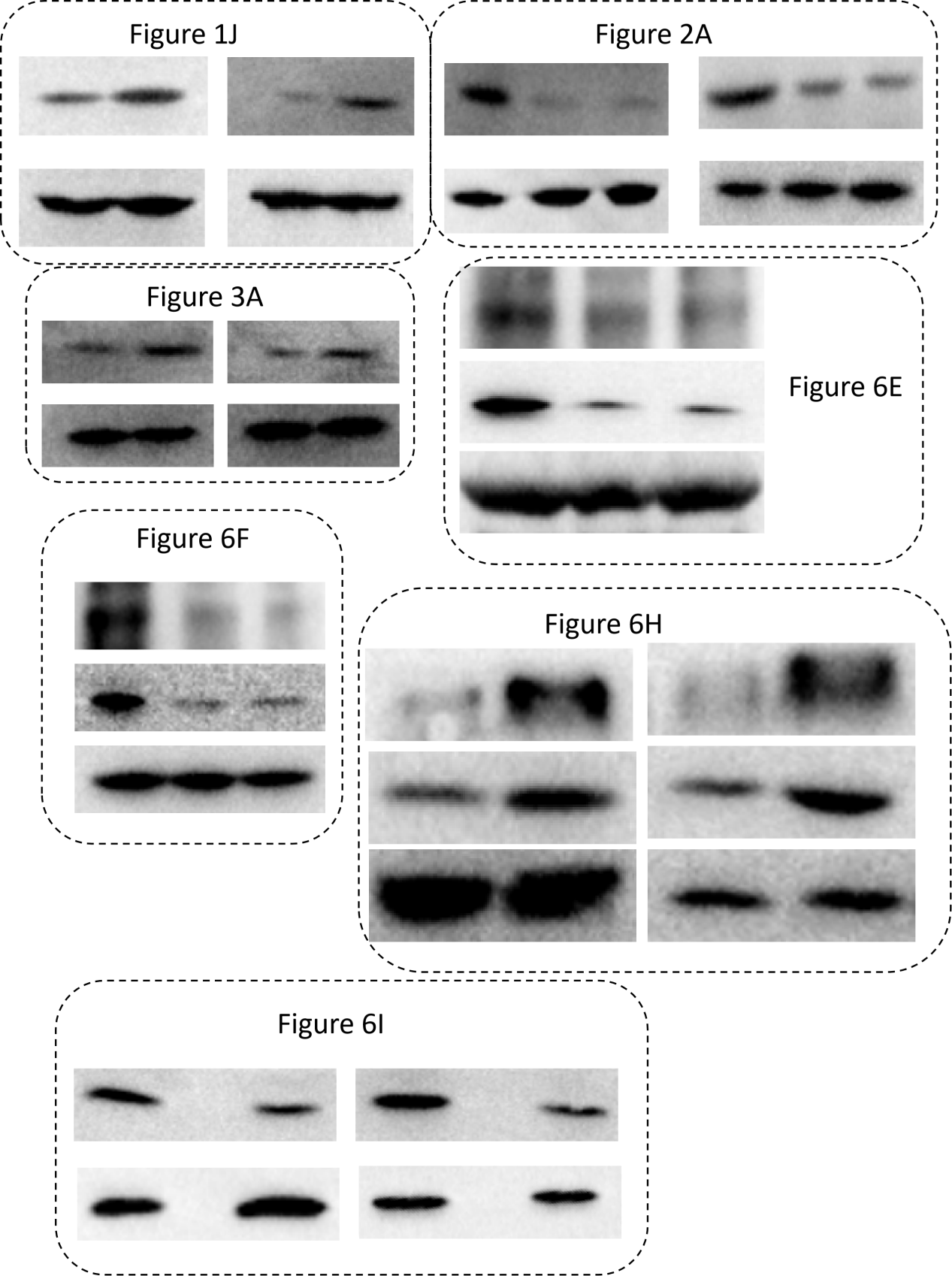


PCR

Figure 5K-5N & Figure S2


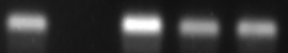

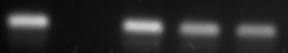

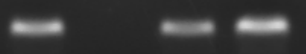

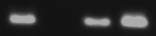

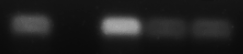

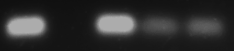

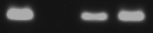

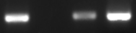

Supplement: Supplementary file 1 — Supplementary Material 1 [file 13402_2026_1227_MOESM1_ESM.docx]
