# Supplementary material for: Targeting RPRD1B overcomes chemoresistance in gastric cancer by suppressing the TOPBP1-mediated DNA damage repair pathway
Source: Cell Oncol (Dordr). 2026 Aug 2;49(3):84. doi: 10.1007/s13402-026-01227-0 (PMC13428727; doi:10.1007/s13402-026-01227-0)
Supplement: Supplementary file 2 — Supplementary Material 2 [file 13402_2026_1227_MOESM2_ESM.docx]

**Supplementary Table. 1 Primer Sequences for RT-qPCR.**

| Gene Name | Sequence | |
| --- | --- | --- |
| RPRD1B-F | | CAGGACCCCTCTTGACTGAG |
| RPRD1B-R | | CGGACAGTAGCATCCCCTGA |
| TOPBP1-F | | TGTGACCCTTTTAGTGGCGTT |
| TOPBP1-R | | CTCTTGGGACACATCGCTGG |
| GAPDH-F | | CTGGGCTACACTGAGCACC |
| GAPDH-R | | AAGTGGTCGTTGAGGGCAATG |

**Supplementary Table. 2 Primer Sequences for ChIP-PCR.**

| Gene Name | Sequence | |
| --- | --- | --- |
| TOPBP1-F | | CAGGACCCCTCTTGACTGAG |
| TOPBP1-R | | AAGTGGTCGTTGAGGGCAATG |

**Supplementary Figure Legends**

**
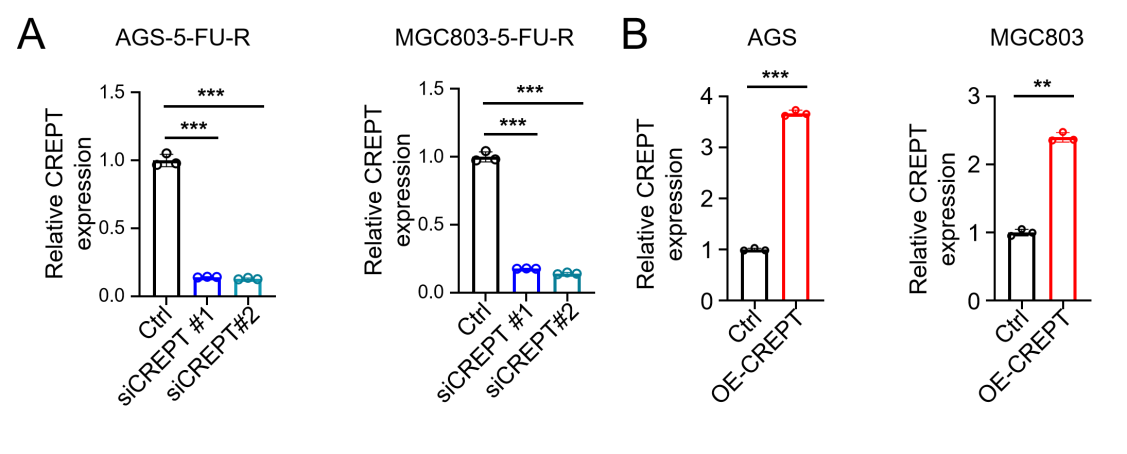
Supplementary Figure 1** CREPT mRNA expression levels were verified/confirmed by RT-qPCR. (A) RT-qPCR were conducted to detect the expression levels of RPRD1B in the control and RPRD1B-knockdown AGS-5-FU-R and MGC803-5-FU-R cells. (B) RT-qPCR were conducted to detect the expression levels of RPRD1B in control and CREPT‑overexpressing AGS and MGC803 cells.


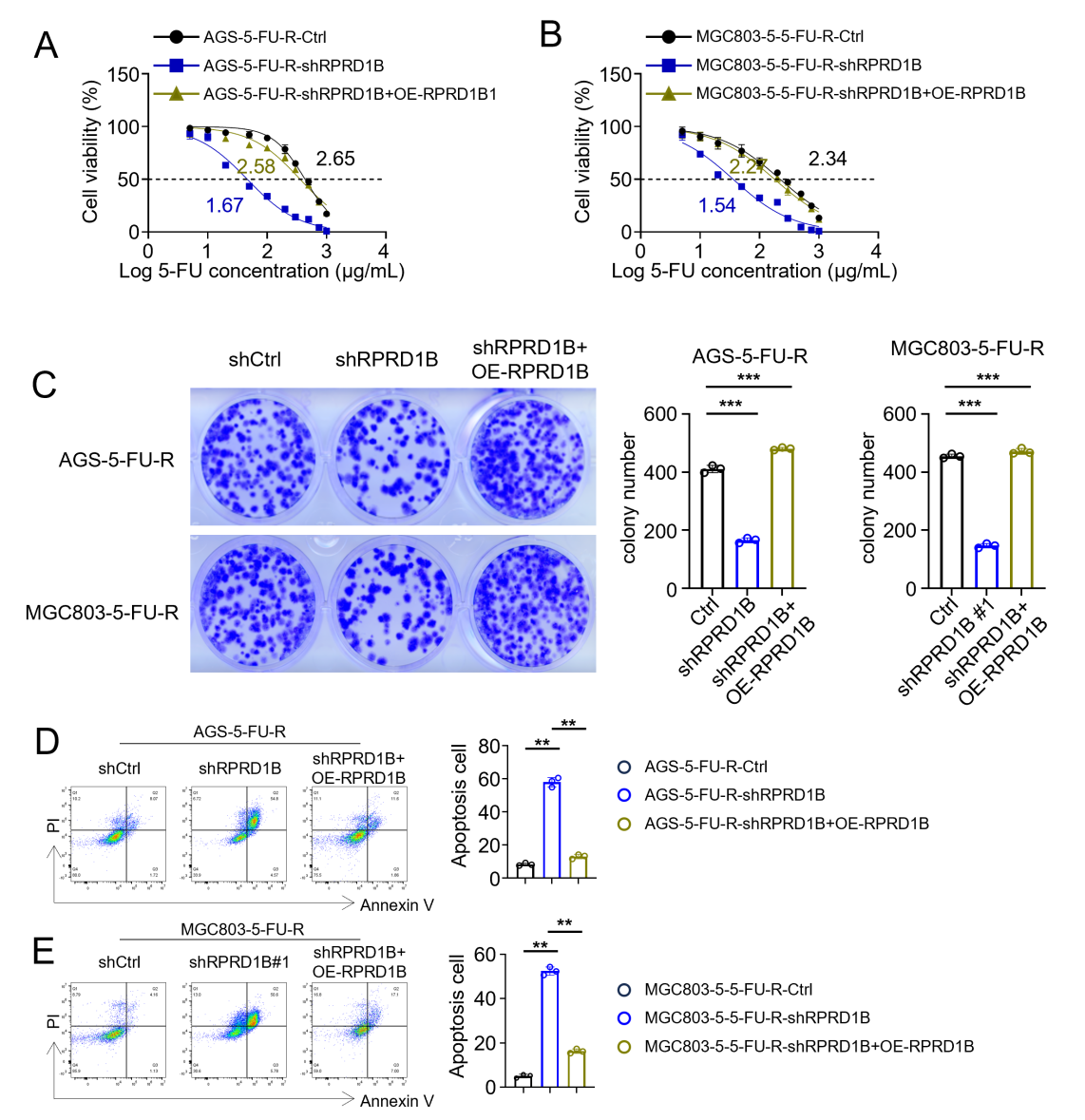


**Supplementary Figure 2** In AGS-5-FU-R and MGC803-5-FU-R cells, we introduced RPRD1B cDNA (OE-RPRD1B) into RPRD1B-depleted cells and assessed the effects on 5-FU sensitivity (A-B), colony formation (C) and apoptosis(D-E).


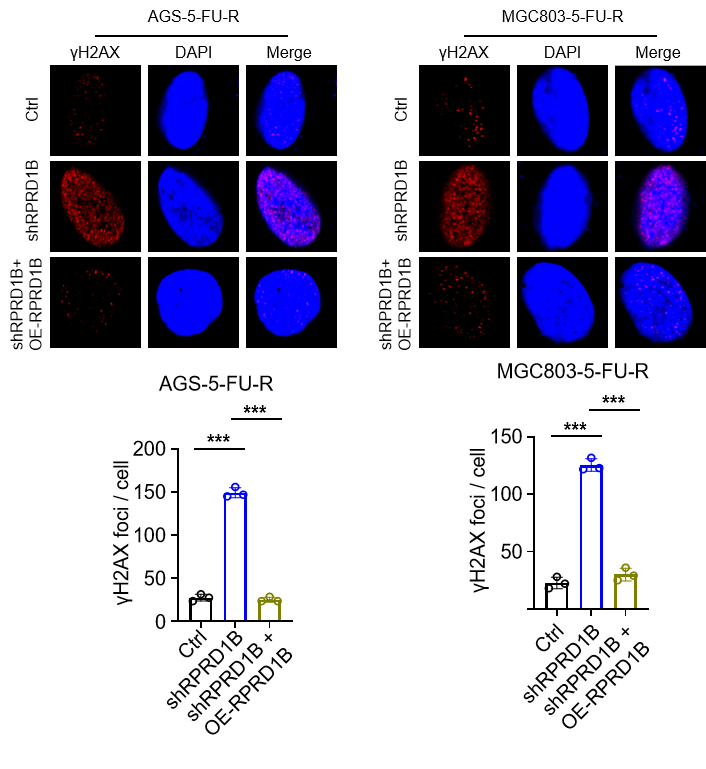


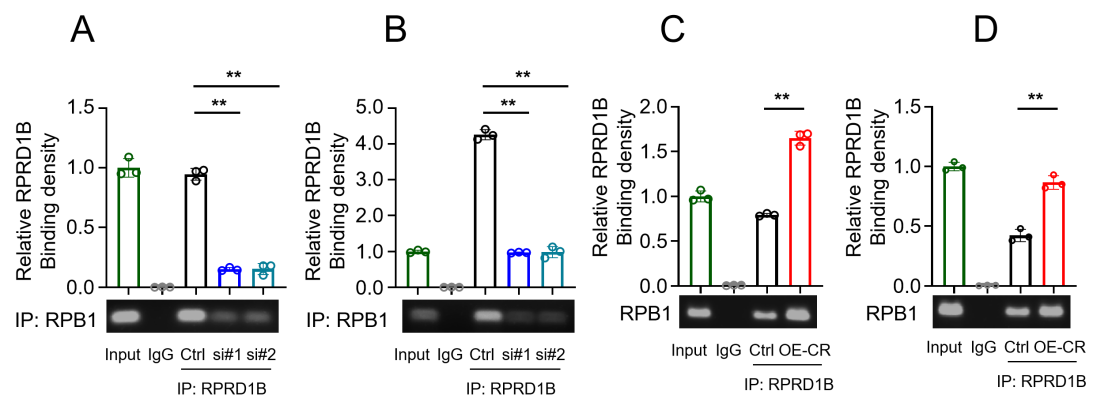
**Supplementary Figure 3** In AGS-5-FU-R and MGC803-5-FU-R cells, we introduced RPRD1B cDNA (OE-RPRD1B) into RPRD1B-depleted cells and assessed the effect of RPRD1B on γH2AX foci formation (A-B).

**Supplementary Figure 4** ChIP‑qPCR analysis of RPRD1B binding at the TOPBP1 promoter. (A‑B) ChIP‑qPCR analysis of RPRD1B binding at the TOPBP1 promoter in control vs. RPRD1B‑knockdown AGS‑5‑FU‑R and MGC803‑5‑FU‑R cells. (C‑D) ChIP‑qPCR analysis of RPRD1B binding at the TOPBP1 promoter in control vs. RPRD1B‑overexpressing AGS and MGC803 cells treated with 5‑FU.


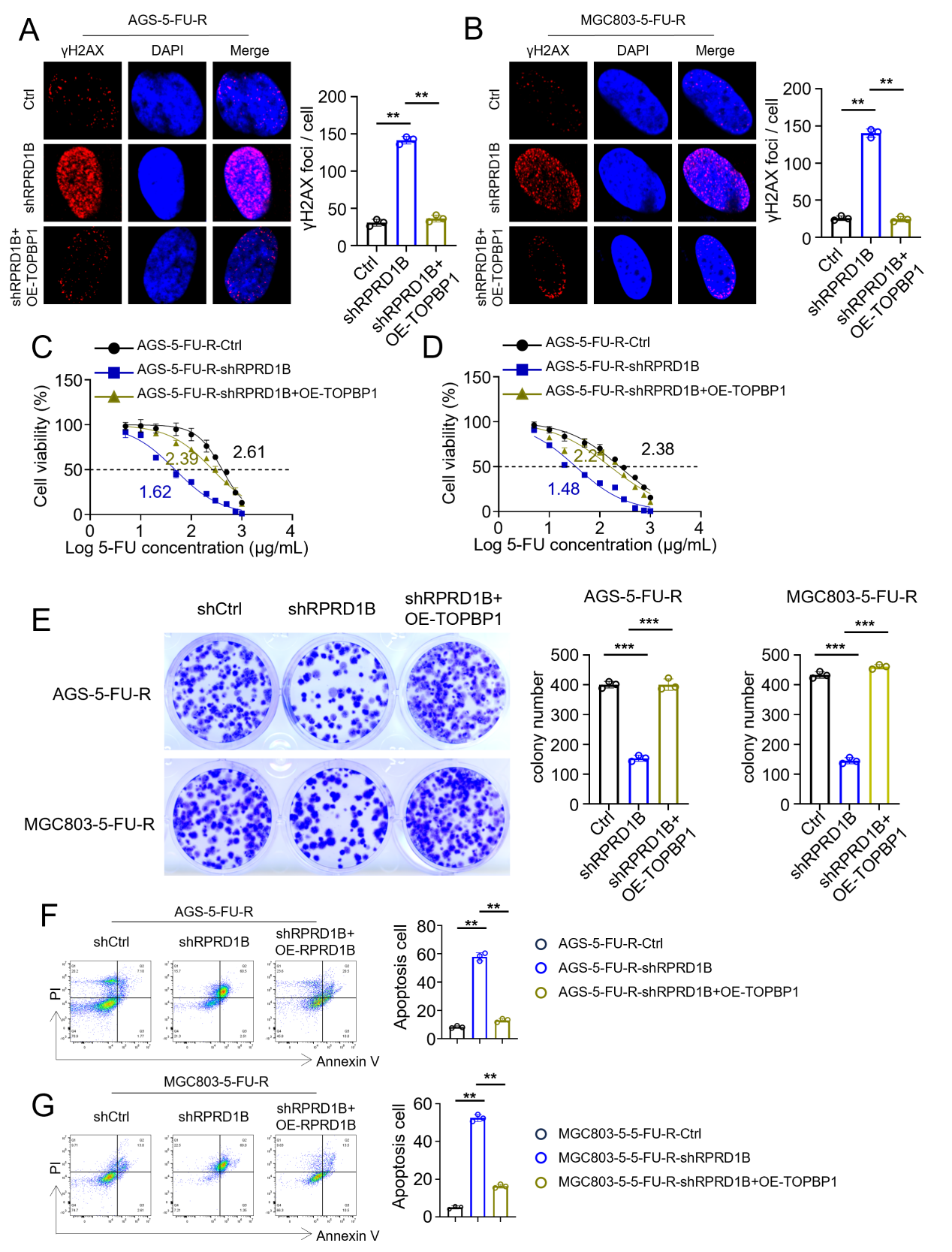


**Supplementary Figure 5** In AGS-5-FU-R and MGC803-5-FU-R cells, we introduced TOPBP1 cDNA (OE-TOPBP1) into RPRD1B-depleted cells and assessed the effects on γH2AX foci formation (A-B), 5-FU sensitivity (C-D), colony formation (E) and apoptosis(F-G).


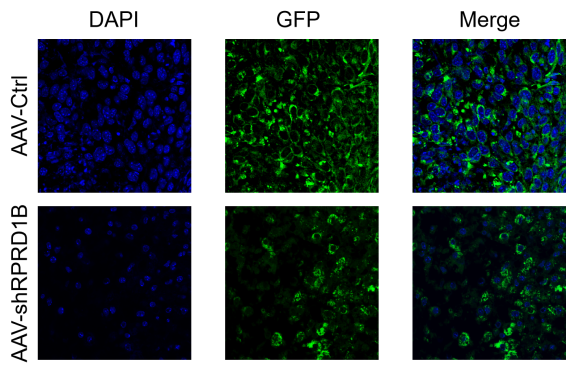


**Supplementary Figure 6** The efficiency of the AAV‑delivered shRNA targeting CREPT (AAV‑Ctrl/shCREPT) was confirmed by GFP immunofluorescence.
